# Training differentially regulates elastin level and proteolysis in skeletal and heart muscles and aorta in healthy rats

**DOI:** 10.1242/bio.017459

**Published:** 2016-04-11

**Authors:** Anna Gilbert, Aleksandra Wyczalkowska-Tomasik, Malgorzata Zendzian-Piotrowska, Bozena Czarkowska-Paczek

**Affiliations:** 1 Department of Clinical Nursing, Medical University of Warsaw, E. Ciolka Street 27, 01-445 Warsaw, Poland; 2 Department of Immunology, Transplantology and Internal Diseases, Medical University of Warsaw, Nowogrodzka Street 59, 02-006 Warsaw, Poland; 3 Department of Physiology, Medical University of Bialystok, Mickiewicza Str. 2C, 15-222 Bialystok, Poland

**Keywords:** Exercise, Proteolytic enzymes, Skeletal muscle, Heart muscle, Aorta, Endurance training

## Abstract

Exercise induces changes in muscle fibers and the extracellular matrix that may depend on elastin content and the activity of proteolytic enzymes. We investigated the influence of endurance training on the gene expression and protein content and/or activity of elastin, elastase, cathepsin K, and plasmin in skeletal and heart muscles and in the aorta. Healthy rats were randomly divided into untrained (*n*=10) and trained (*n*=10; 6 weeks of endurance training with increasing load) groups. Gene expression was evaluated via qRT-PCR. Elastin content was measured via enzyme-linked immunosorbent assay and enzyme activity was measured fluorometrically. Elastin content was significantly higher in skeletal (*P*=0.0014) and heart muscle (*P*=0.000022) from trained rats versus untrained rats, but not in the aorta. Although mRNA levels in skeletal muscle did not differ between groups, the activities of elastase (*P*=0.0434), cathepsin K (*P*=0.0343) and plasmin (*P*=0.000046) were higher in trained rats. The levels of cathepsin K (*P*=0.0288) and plasminogen (*P*=0.0005) mRNA were higher in heart muscle from trained rats, but enzyme activity was not. Enzyme activity in the aorta did not differ between groups. Increased elastin content in muscles may result in better adaption to exercise, as may remodeling of the extracellular matrix in skeletal muscle.

## Abstract (precis)

**Summary:** Endurance training increases elastin content in muscles but not in the aorta. The activities of enzymes responsible for ECM remodeling increase only in skeletal muscle. These changes seem to be adaptive.

## INTRODUCTION

Physical activity, particularly endurance training, causes many adaptive changes in the organism. These adaptations mainly occur in skeletal muscles and include changes in metabolism and tissue composition (Röckl et al., 2007). Adaptive changes in the extracellular matrix (ECM) occur at the same time. ECM not only provides scaffolding and structural support for cells and organs, it also exchanges information with cells and thereby modulates cellular development, attachment, and differentiation as well as tissue repair (Hayden et al., 2005; Fonović and Turk, 2014). ECM remodeling in skeletal muscle influences cellular processes including DNA synthesis, microtubule fragmentation, and myoblast fusion (Calve et al., 2010), all of which improve muscle strength and render tissue more compliant and resistant to damage (Hayden et al., 2005). The ECM is also involved in the regeneration of muscle fibers (Suelves et al., 2002). Elastase, cathepsin K, and plasmin contribute to the remodeling of ECM components, including elastin (Antonicelli et al., 2007), which is mainly responsible for tissue elasticity (Boudoulas et al., 2012); inhibition of ECM-modifying enzymes previously resulted in aberrant muscle regeneration (Vinarsky et al., 2005). Proteolytic enzymes may also directly influence muscle fibers, for instance by inducing apoptosis (Doeuvre et al., 2010).

The aim of this study was to investigate the influence of 6 weeks of endurance training on the mRNA levels of *tropoelastin*, *elastase*, *cathepsin K* and *plasminogen* in skeletal muscle (soleus) and heart muscle (ventricle) from healthy rats. We also characterized the effect of training on elastin protein levels and the activities of elastase, cathepsin K, and plasmin in muscles and the aorta.

## RESULTS

**Fig. 1.**
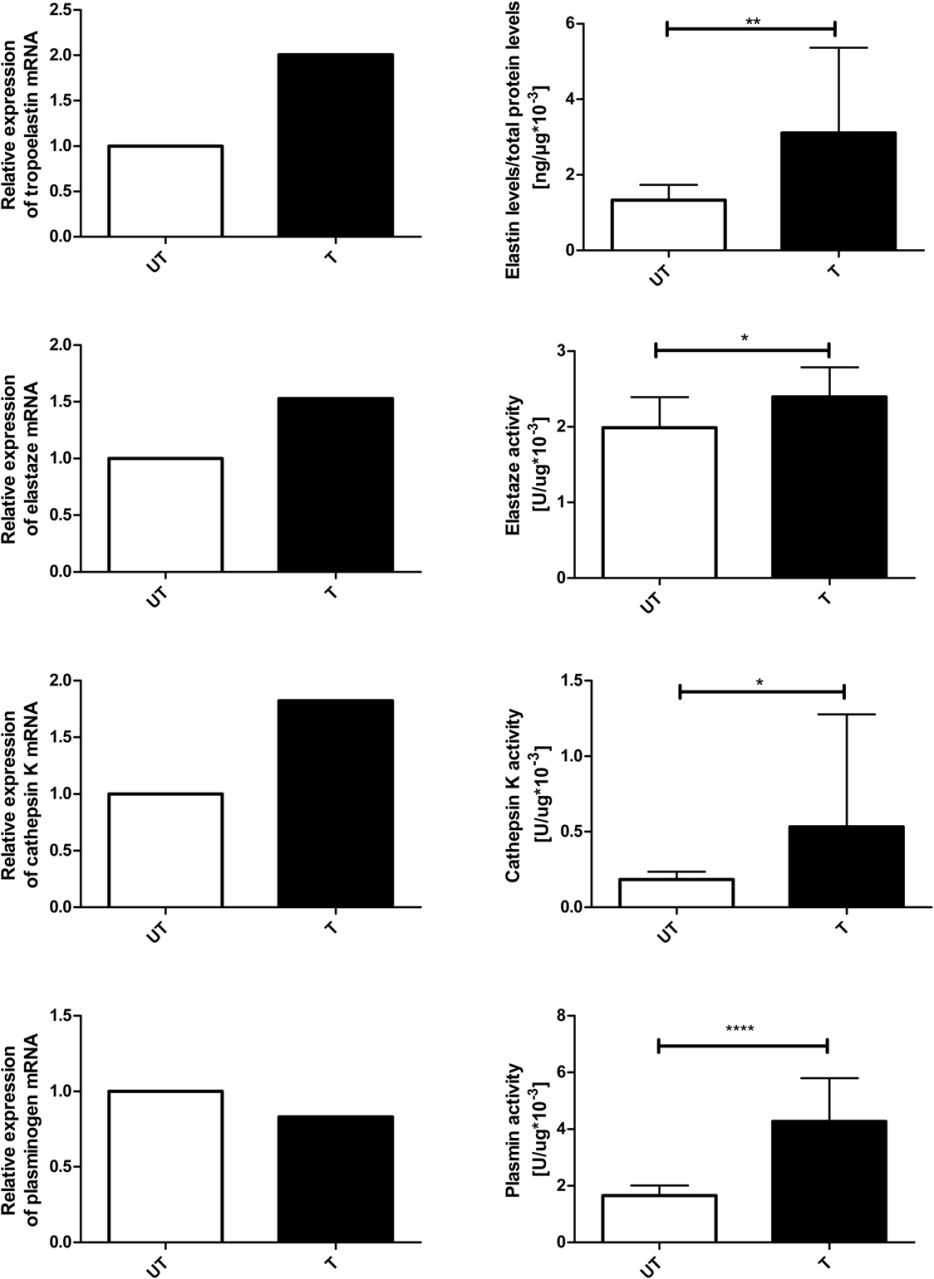
**Effect of endurance training on gene expression, and protein content and activity in soleus muscle.** mRNA levels of *tropoelastin* (UT, *n*=10; T, *n*=10), *elastase* (UT, *n*=9; T, *n*=9), *cathepsin K* (UT, *n*=10; T, *n*=10), and *plasminogen* (UT, *n*=10; T, *n*=9) did not differ significantly between trained and untrained rats. Elastin protein concentrations (UT, *n*=10; T, *n*=8) were significantly higher in trained rats than in untrained rats (*P*=0.0014). The activities of elastase (UT, *n*=10; T, *n*=8; *P*=0.0434), cathepsin K (UT, *n*=10; T, *n*=8; *P*=0.0343), and plasmin (UT, *n*=10; T, *n*=8; *P*=0.000046) were significantly higher in trained rats than in untrained rats. The experiments were performed in duplicates, except for elastin protein concentration which was made in single repetition. Error bars express s.d. Mann–Whitney test was used for comparisons. *P≤0.05; **P≤0.01; ****P≤0.0001.

In skeletal muscle (soleus), the mRNA levels of tropoelastin [untrained (UT), *n*=10; trained (T), *n*=10], elastase (UT, *n*=9; T, *n*=9), cathepsin K (UT, *n*=10; T, *n*=10), and plasminogen (UT, *n*=10; T, *n*=9) did not differ significantly between trained and untrained rats (Fig. 1). However, elastin protein concentrations (UT, *n*=10; T, *n*=8) were significantly higher in trained rats than in untrained rats (*P*=0.0014; Fig. 1). The activities of elastase (UT, *n*=10; T, *n*=8; *P*=0.0434), cathepsin K (UT, *n*=10; T, *n*=8; *P*=0.0343), and plasmin (UT, *n*=10; T, *n*=8; *P*=0.000046) were significantly higher in trained rats than in untrained rats (Fig. 1).

**Fig. 2.**
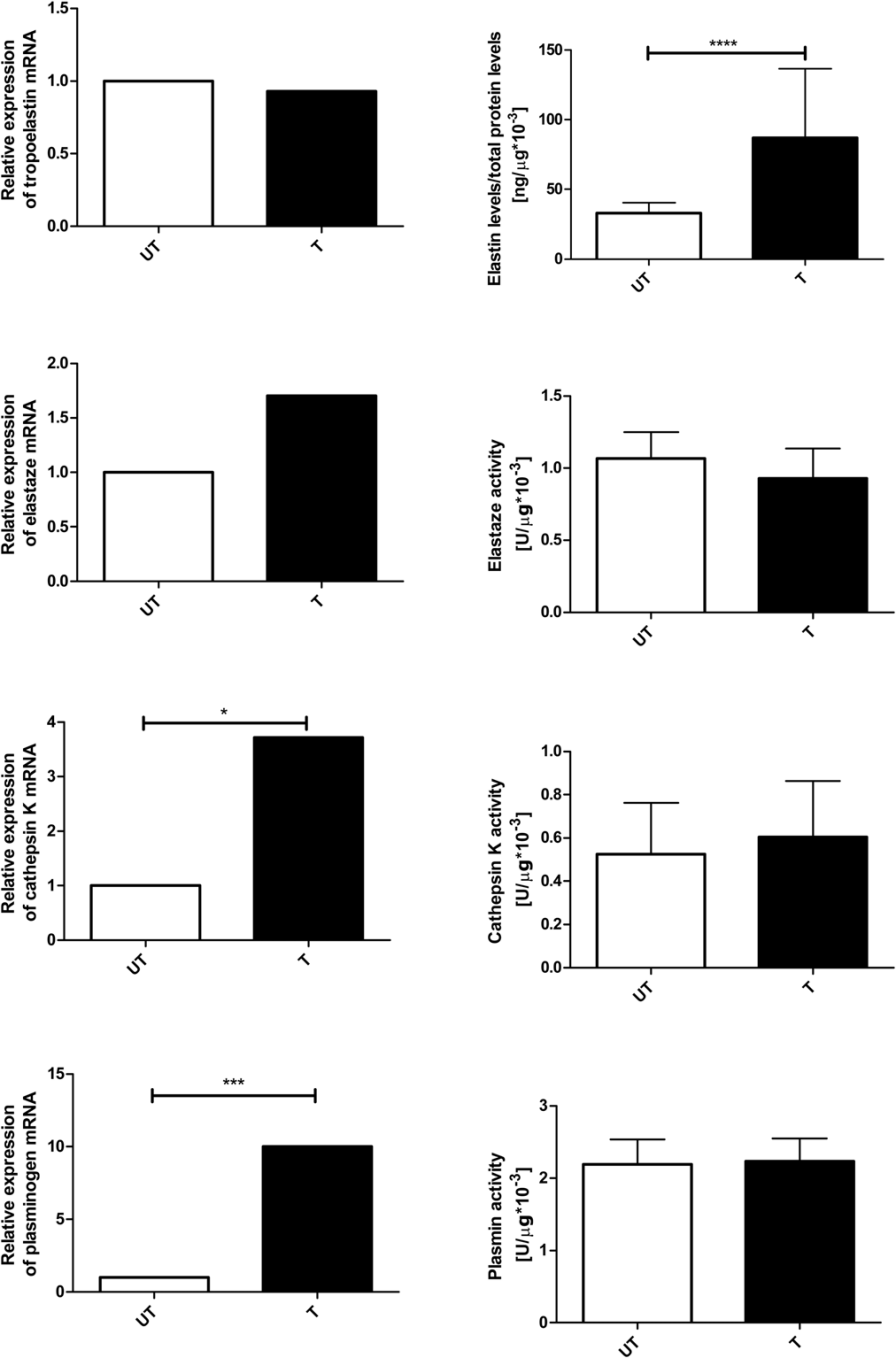
**Effect of endurance training on gene expression, and protein content and activity in heart muscle.** The mRNA levels of *cathepsin K* (UT, *n*=10; T, *n*=10; *P*=0.0288) and *plasminogen* (UT, *n*=10; T, *n*=10; *P*=0.0005) were higher in the heart muscle (ventricle) of trained rats than in untrained rats. There were no significant between-group differences in the mRNA levels of *tropoelastin* (UT, *n*=10; T, *n*=10) and *elastase* (UT, *n*=10; T, *n*=10). Elastin protein concentrations (UT, *n*=10; T, *n*=9; *P*=0.000022) were significantly higher in trained rats than in untrained rats. The activities of proteolytic enzymes did not differ between groups (UT, *n*=10; T, *n*=10). The experiments were performed in duplicates, except for elastin protein concentration which was made in single repetition. Error bars express s.d. Mann–Whitney test was used for comparisons. *P≤0.05; ***P≤0.001; ****P≤0.0001.

The mRNA levels of cathepsin K (UT, *n*=10; T, *n*=10; *P*=0.0288) and plasminogen (UT, *n*=10; T, *n*=10; *P*=0.0005) were higher in the heart muscle (ventricle) of trained rats than in this muscle in untrained rats (Fig. 2). Although there were no significant between-group differences in the mRNA levels of tropoelastin (UT, *n*=10; T, *n*=10) and elastase (UT, *n*=10; T, *n*=10; Fig. 2), elastin protein concentrations were significantly higher in trained rats than in untrained rats (UT, *n*=10; T, *n*=9; *P*=0.000022; Fig. 2). The activities of proteolytic enzymes did not differ between groups (UT, *n*=10; T, *n*=10; Fig. 2).

**Fig. 3.**
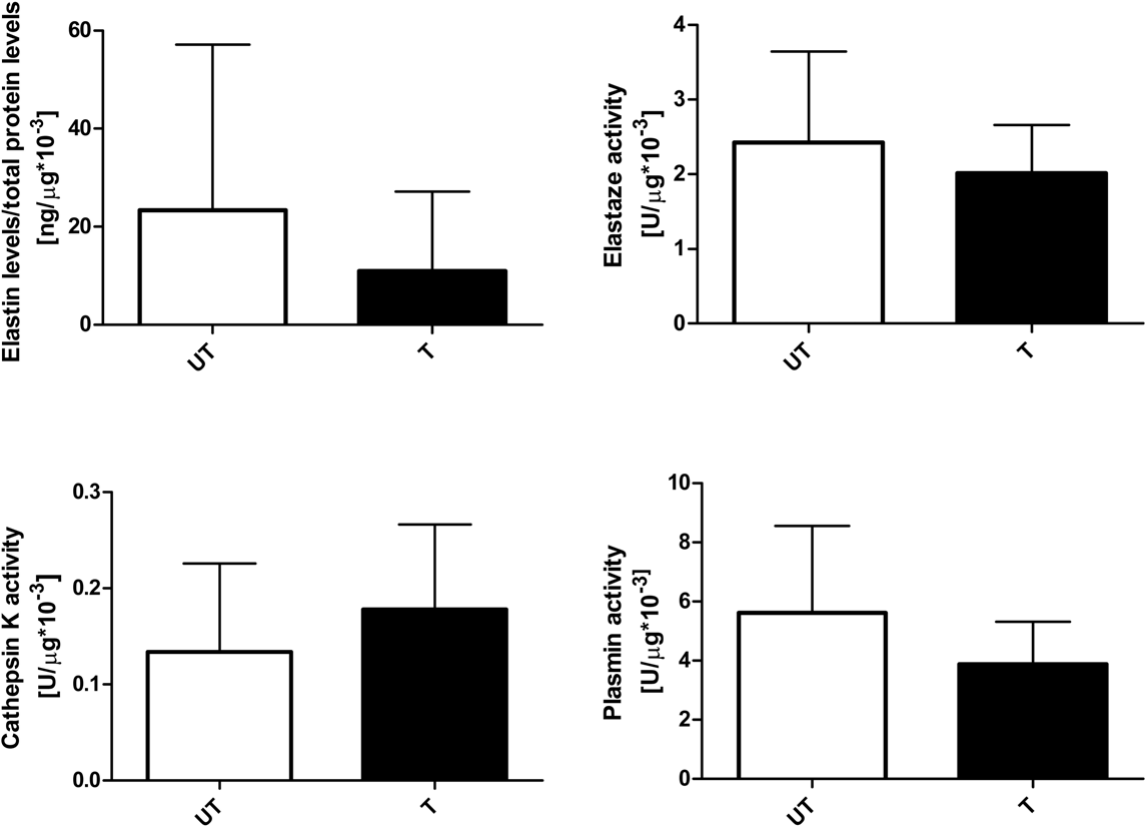
**Effect of endurance training on protein content and activity in aorta.** There were no significant differences in elastin content (UT, *n*=10; T, *n*=7) or the activities of the proteolytic enzymes elastase (UT, *n*=10; T, *n*=10), cathepsin K (UT, *n*=9; T, *n*=10), and plasmin (UT, *n*=10; T, *n*=10) in trained rats versus untrained rats. The experiments were performed in duplicates, except for elastin protein concentration which was made in single repetition. Error bars express s.d. Mann–Whitney test was used for comparisons.

We did not measure mRNA levels in aorta samples due to the small amounts of available material. In the aorta, there were no significant differences in elastin content (UT, *n*=10; T, *n*=7) or the activities of the proteolytic enzymes elastase (UT, *n*=10; T, *n*=10), cathepsin K (UT, *n*=9; T, *n*=10), and plasmin (UT, *n*=10; T, *n*=10) in trained rats versus untrained rats (Fig. 3).

All results are presented as medians with min and max in Table 1.

**Table 1.**
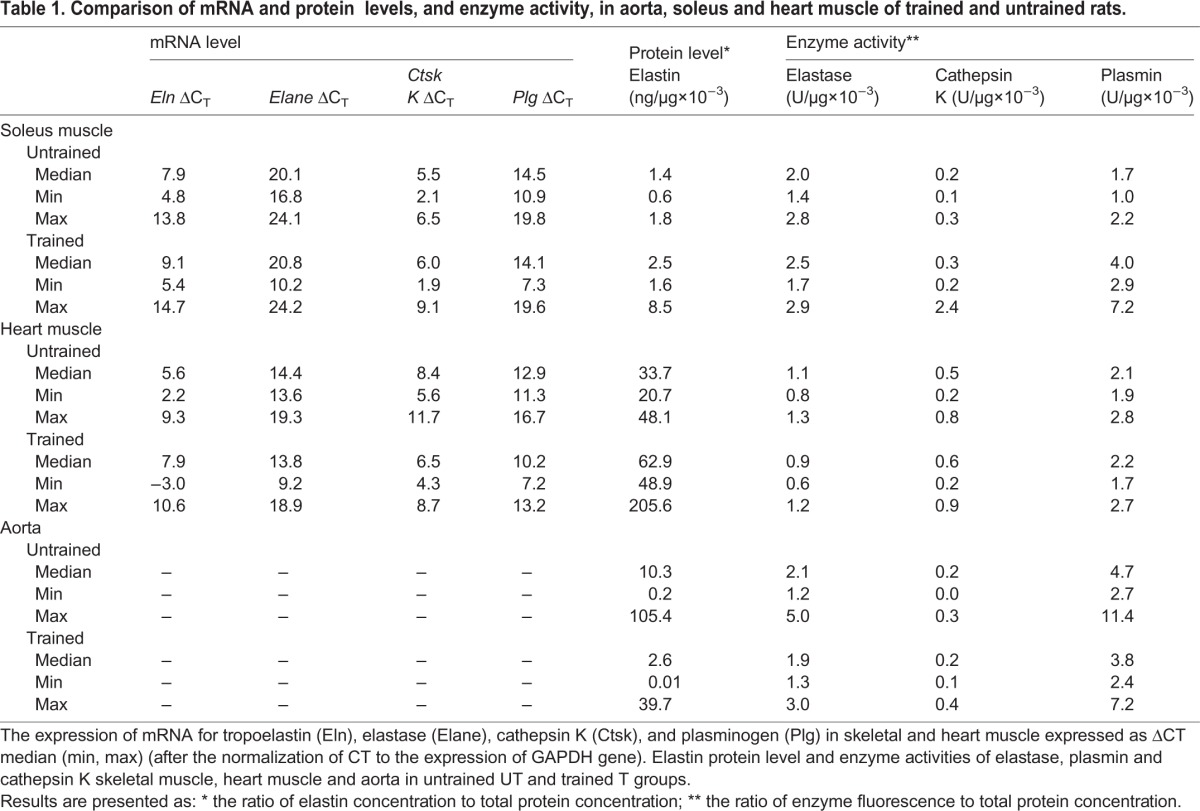
**Comparison of mRNA and protein levels, and enzyme activity, in aorta, soleus and heart muscle of trained and untrained rats.**

## DISCUSSION

The principal finding of this study is that endurance training differentially modulates elastin mRNA and protein content as well as the mRNA expression and activity of proteolytic enzymes in a tissue-dependent manner. Here, skeletal and heart muscle exhibited similar adaptive changes in elastin expression after training; gene expression did not differ between groups, but elastin protein levels were higher in trained rats than in untrained rats. Post-transcriptional modifications may underlie this differential response. In mammalian cells, the correlation coefficient between mRNA and protein levels was previously determined to be <0.5 (Pradet-Balade et al., 2001).

Elastin levels may influence the elastic and force-bearing features of the ECM (Lehti et al., 2006). Heart muscle contains few elastic fibers; its physiological compliance stems mainly from cardiomyocytes (Mizuno et al., 2005). Nonetheless, in the myocardial ECM, elastin makes important contributions to the maintenance of structural integrity, the transmission of mechanical stress into and out of myocardial cells, elasticity and compliance during the cardiac cycle, and the prevention of excessive stretching (Kwak et al., 2011).

There are some investigations addressing the influence of physical exercise on elastin mRNA and protein levels in skeletal muscle. Lehti et al. showed that endurance training reversed decreases in elastin transcription in skeletal muscle from diabetic mice but in accordance with the present study, elastin mRNA levels were not affected by training in healthy mouse and sedentary healthy controls (Lehti et al., 2006). Additionally, few studies have evaluated elastin expression and protein content in the heart, and these studies mainly focused on heart failure. Consistent with our observations, (Marshall et al., 2013) reported that relative elastin mRNA levels did not significantly differ between Yucatan miniature swine with induced heart failure that exercised versus those that remained sedentary (both healthy control and sedentary with heart failure). In our study, despite similarity at the level of gene expression, elastin protein levels were higher in our trained rats than in our untrained rats, which may reflect an adaptive mechanism in healthy subjects that affects force transmission and the resistance to injury of skeletal muscle after physical training (McHugh, 2003). In heart muscle, this mechanism may contribute to the well-known increase in heart compliance after training (Stickland et al., 2006). The specific roles of elastin in skeletal and heart muscle are not well described in the literature (Fomovsky et al., 2010).

In the present study, post-training changes in proteolytic enzymes differed between skeletal muscle and heart muscle. In skeletal muscle, the mRNA levels of the investigated enzymes were similar in trained and untrained rats, but the activities of elastase, cathepsin K, and plasmin were significantly higher in trained rats than in untrained rats. In heart muscle, the mRNA levels of cathepsin K and plasminogen were higher in trained rats than in untrained rats, but the activities of these enzymes did not differ between groups. The discrepancy between gene expression and enzyme activity observed here may stem from the low coefficient of correlation between mRNA levels and protein levels in mammalian cells (Pradet-Balade et al., 2001). This discrepancy also suggests the presence of a post-translational mechanism and perhaps other mechanisms that influence enzyme activity. For example, numerous studies have reported decreased activity of plasminogen activator inhibitor-1 in plasma after training (Jahangard et al., 2009).

The roles of elastase, cathepsin K, and plasmin in the adaptation of skeletal muscle to physical exercise are unclear. It is worth mentioning that in our study, proteolytic activity in skeletal muscle coincided with increased elastin levels in the soleus muscle of trained rats, indicating that adaptation does not translate into lower elastin content in soleus muscle.

The elastases belong to the group of serine, metallo-, or cysteine proteases. They degrade elastin and several matrix and non-matrix substrates such as fibronectin, laminin, collagen (types III, IV, and VI), and proteoglycans (Antonicelli et al., 2007; Paczek et al., 2008). While there is little data on the influence of physical training on the generation of elastase in skeletal muscle, single bouts of physical activity are known to increase elastase (Serteyn et al., 2010; Gleeson et al., 1998). Elastase content remained increased in triathletes as long as 19 days after the race (Neubauer et al., 2008).

Cathepsin K belongs to the family of lysosomal cysteine cathepsins; it is involved in the turnover of ECM proteins in many organs, and contributes to cardiovascular disease (including atherosclerosis and aortic aneurysms), inflammation, and obesity (Lv et al., 2013; Podgorski, 2009). In addition, cathepsin K may be a collagenase (Antonicelli et al., 2007) and may play a role in the prevention of muscle fibrosis.

Plasmin mediates blood-clot dissolution and is necessary for myogenesis, muscle regeneration, and hypertrophy (Suelves et al., 2002; López-Alemany et al., 2003). It can degrade several ECM proteins either directly or by activating matrix metalloproteinases 1-3 or 9. Plasmin also drives the inflammatory response (Syrovets and Simmet, 2004; Li et al., 2007). Plasmin may prevent intramuscular fibrin accumulation and contribute to an accurate inflammatory response in muscles after injury (Lluís et al., 2001).

Given these previous reports, we conclude that all of the enzymes evaluated in the present study take part in ECM remodeling and that ECM in skeletal muscle plays a very important role in providing tissue with elastic properties, giving mechanical support to myofibers during muscle contractions, and participating in the transmission of force from myofibers to tendons (Lehti et al., 2006). Additionally, extracellular proteolysis is necessary for the development and regeneration of skeletal muscle. The adaptation of muscle to physical exercise is a complex process that relies, at least in part, on the increased local proteolytic activity observed in the present study. However, we note that despite concomitant increases in gene expression, the lack of change in proteolytic activity in heart muscle that was detected here indicates that adaptation does not take place in heart muscle.

In our study, there were no significant differences in elastin content and enzyme activity in the aorta of trained versus untrained rats. Such results are in line with the results obtained by others. For example, 8 weeks of aerobic training had no effect on aortic elastin content in 6-month-old normotensive rats (Niederhoffer et al., 2000); another study failed to uncover a difference in elastin content between trained rats and sedentary controls (both young and old) after 17-21 weeks of swimming training (Nosaka et al., 2003). Similarly, no training effect occurred in a voluntary running group (Matsuda et al., 1989; Matsuda et al., 1993). Training-induced increases in elastin levels were previously observed in aged mice or hypertensive rats (Moraes-Teixeira et al., 2010; Kadoglou et al., 2011). However, spontaneously hypertensive rats exhibited higher mRNA levels of elastin and markedly higher elastin/collagen content; training effectively corrected the elastin content in the aorta of these hypertensive rats, reducing pulsatility, facilitating buffering, and reducing cardiovascular risk (Jordão et al., 2011). Overall, most previous studies described differences in the elastin content of the aorta in the context of existing pathology or aging, but not in healthy subjects.

### Conclusions

Our results indicate that endurance training activates different signaling pathways in various tissues. Increased elastin content may translate into increased compliance; we detected this increase in heart and skeletal muscle but not in the aorta. The activities of enzymes responsible for ECM remodeling increase in skeletal muscle and may function in concert with the adaptation of skeletal muscle to physical training, mainly by this mechanism, but also via direct effects on muscle cells. Such a mechanism was not evident in heart muscle or in the aorta in the present investigation.

## MATERIALS AND METHODS

All procedures used in this study were approved by the Ethical Committee of the Medical University in Bialystok, Poland (Resolution No. 23/2011 on the proposal No./dated 27.04.2011) and were performed in accordance with European Union regulations regarding the humane treatment of laboratory animals.

Twenty male Wistar rats were used in this study. The rats had *ad libitum* access to water and were fed with Labofeed B under a 12 h light/12 h dark cycle. For the first 5 days, rats were subjected to exercise adaptation via a once-daily regime of 10 min of running on a treadmill at 15 m/min. Rats were then randomly assigned to one of two groups: untrained (UT, *n*=10) or trained (T, *n*=10). Rats in the trained group were subjected to exercise training 5 days per week for 6 weeks. Exercise intensity and duration were gradually increased over time. Initially, sessions lasted 10 min (1200 m/h); this duration was increased 10 min each day during the first week for a final duration of 60 min/day, which was maintained over the rest of the training period. The running speed was 1500 m/h in the second week and 1680 m/h for weeks 3-6. There was no additional running stimulation. The untrained group remained sedentary throughout the training period. The age of the rats at the beginning of exercise was 5–6 weeks.

Twenty-four hours after the last training session, all rats were sacrificed under anesthesia (intraperitoneal chloral hydrate, 1 ml/100 mg body mass). The average body mass of rats on the day of sacrifice was 271±11.6 g in the untrained group and 283.17±24.67 g in the trained group. Samples of soleus muscle, heart muscle (ventricle), and aorta were collected and immediately stored at −80°C. Soleus muscle was chosen because it contains a large proportion of type I slow-twitch fibers (Feng et al., 2011). Soleus muscle is primarily recruited during running at the speeds used in our study, while fast-twitch muscles generally are not (Lambert and Noakes, 1989).

We measured the mRNA levels of *tropoelastin*, *elastase*, *cathepsin K* and *plasminogen* in skeletal and heart muscle. Tropoelastin is a soluble precursor of elastin (Vrhovski and Weiss, 1998) and plasminogen is the inactive precursor of plasmin (Novokhatny, 2008). We also evaluated elastin protein content as well as the activities of elastase, cathepsin K, and plasmin in both muscle types. Only elastin protein content and the activity of proteolytic enzymes were investigated in samples from the aorta due to the small amount of available material.

### Total RNA isolation

Approximately 50 mg of heart muscle (ventricle) or soleus muscle were homogenized in QIAZOL (Qiagen, Germany) plus 8 µl proteinase K (Qiagen) in a TissueLyser bead mixer (Qiagen) at 25 Hz in two 5-min repetitions. Total RNA isolation was performed with an EZ1 RNA Universal Tissue Kit and Biorobot EZ1 (Qiagen) in accordance with the manufacturer's instructions. Total RNA concentrations were measured at 260 nm via spectrophotometry (ND-1000 spectrophotometer, NanoDrop Technologies, Inc.). Samples were frozen and stored at −80°C for subsequent analysis.

### Quantitative reverse transcription polymerase chain reaction (qRT-PCR)

mRNA levels were measured with the ABI-Prism 7500 Sequence Detection System (Applied Biosystems, USA). Specific probes and primers for rat *glyceraldehyde 3-phosphate dehydrogenase* (Assay ID: Rn01775763_g1), *tropoelastin* (Assay ID: Rn01499782_m1), *neutrophil elastase* (Assay ID: Rn01535456_g1), *cathepsin K* (Assay ID: Rn00580723_m1) and *plasminogen* (Assay ID: Rn00585167_m1) and the TaqMan One-Step RT-PCR Master Mix Reagents Kit were purchased from Applied Biosystems.

mRNA levels were calculated using the comparative cycle threshold (C_T_) method. The C_T_ of each sample was normalized to the expression of *glyceraldehyde 3-phosphate dehydrogenase* (*GAPDH*), with results reported as ΔC_T_. According to Pérez et al. *GAPDH* is optimal gene to be used as reference gene in the heart (Pérez et al., 2007). The relative mRNA levels of the investigated proteins were calculated by subtracting the normalized C_T_ values for the trained group relative to the median untrained value (ΔΔC_T_=ΔC_T_, trained−ΔC_T_,untrained), and the relative fold change of the mRNA levels of the investigated proteins was calculated as 2^−ΔΔCT^ (Livak and Schmittgen, 2001).

### Tissue homogenization and total protein quantification

Due to the limited amount of sample, homogenization of each sample was performed as follows. All samples were homogenized in water in a TissueLyser bead mixer (Qiagen) and centrifuged twice at 7826 ***g*** for 10 min at 4°C. Plasmin activity and elastase activity were assayed directly after centrifugation. Supernatants were stored at −80°C for further analyses of cathepsin K, elastin, and total protein content.

For the determination of elastin levels, samples of heart muscle were homogenized in phosphate-buffered saline in accordance with the manufacturer's (see below) instructions and stored overnight at −20°C. After two freeze-thaw cycles, the homogenates were centrifuged for 5 min at 5000 ***g***. The supernatant was removed and assayed immediately as described below.

Total protein concentration was measured at 562 nm on a BioTek Power Wave XS spectrophotometer (BioTek Instruments, USA) using the bicinchoninic acid Protein Assay Reagent (Pierce, Holland) in accordance with the manufacturer's instructions.

### Quantification of elastin levels

Elastin levels were measured in tissue homogenates via enzyme-linked immunosorbent assay (ELISA). Concentrations were measured at 562 nm on a BioTek Power Wave XS spectrophotometer using the Elastin ELISA Kit (EiAab, China). Results are presented as the ratio of elastin concentration to total protein concentration.

### Assays of enzyme activity

Enzyme activity was measured using a spectrofluorimeter (LS-50B, PerkinElmer, USA). Fluorescence measurements were made with induction at λ=355 nm and emission at λ=460 nm. The substrate for elastase was Z-Arg-Arg-7-amido-4-methylcoumarin and the substrate for plasmin was Boc-Val-Leu-Lys-7-amido-4-methylcoumarin (Bachem, Biochemica GmbH, Germany). A commercial kit (Cathepsin K Activity Fluorometric Assay Kit, BioVision, Inc., USA) was used to measure cathepsin K activity (substrate Ac-Lys-Arg- amino-4-trifluoromethyl coumarin) with a 400-nm excitation filter and a 505-nm emission filter. Results are presented as the ratio of enzyme fluorescence to total protein concentration.

### Statistical analyses

Results are reported as medians with min and max, as mean±standard deviation (s.d.) and as relative fold changes. Differences in mRNA levels (for statistics, ΔC_T_ was used) and protein levels between groups were analyzed with the Mann–Whitney *U*-test. *P*-values <0.05 were considered to be statistically significant.
